# Activated desorption at heterogeneous interfaces and long-time kinetics of hydrocarbon recovery from nanoporous media

**DOI:** 10.1038/ncomms11890

**Published:** 2016-06-21

**Authors:** Thomas Lee, Lydéric Bocquet, Benoit Coasne

**Affiliations:** 1 MultiScale Materials Science for Energy and Environment, Joint CNRS-MIT Laboratory, UMI CNRS 3466, Massachusetts Institute of Technology, Cambridge, Massachusetts 02139, USA; 2 Department of Civil and Environmental Engineering, Massachusetts Institute of Technology, Cambridge, Massachusetts 02139, USA; 3 Laboratoire de Physique Statistique, UMR CNRS 8550, Ecole Normale Supérieure, 75005 Paris, France; 4 Laboratoire Interdisciplinaire de Physique, CNRS and Université Grenoble Alpes, UMR CNRS 5588, 38000 Grenoble, France

## Abstract

Hydrocarbon recovery from unconventional reservoirs (shale gas) is debated due to its environmental impact and uncertainties on its predictability. But a lack of scientific knowledge impedes the proposal of reliable alternatives. The requirement of hydrofracking, fast recovery decay and ultra-low permeability—inherent to their nanoporosity—are specificities of these reservoirs, which challenge existing frameworks. Here we use molecular simulation and statistical models to show that recovery is hampered by interfacial effects at the wet kerogen surface. Recovery is shown to be thermally activated with an energy barrier modelled from the interface wetting properties. We build a statistical model of the recovery kinetics with a two-regime decline that is consistent with published data: a short time decay, consistent with Darcy description, followed by a fast algebraic decay resulting from increasingly unreachable energy barriers. Replacing water by CO_2_ or propane eliminates the barriers, therefore raising hopes for clean/efficient recovery.

## Abstract (web summary)

Hydrocarbon recovery from gas shale challenges existing frameworks for fluid transport due to the nanoporous nature of the shale. Here, the authors use molecular simulation and statistical models to show that gas recovery is thermally activated due to interfacial effects at the nanoporous surface.

## Graphical abstract

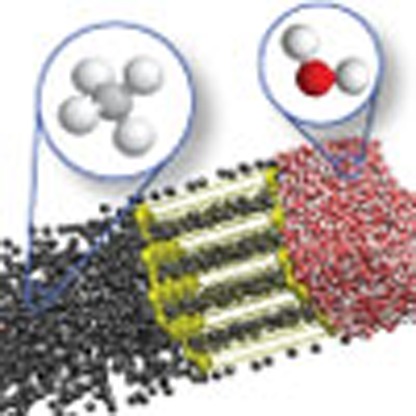

Despite its increasing role in today's energy market, hydrocarbon extraction from gas shale remains poorly understood with many specificities left unexplained. Owing to their ultra-low permeability, typically six orders of magnitude below that of conventional reservoirs, gas and oil recovery from these unconventional reservoirs requires severe stimulation techniques such as hydrofracking. Moreover, different wells display a broad, unexpected variability in hydrocarbon production, which rapidly declines over several months, typically algebraically in time^2^,^3^,^4^,^5^,^6^,^7^. Typically, shale gas reservoirs consist of a collection of kerogen pockets, the host nanoporous organic material containing the hydrocarbons, distributed throughout the mineral shale rock (sketched in Fig. 1)^8^. On hydraulic fracturing, these nanoporous kerogen reservoirs connect to the macroscopic fracture network and release their hydrocarbon content as the pressure in the fracking fluid is decreased. This picture strongly differs from standard oil recovery from conventional reservoirs, which is usually described within the framework of fluid dynamics in porous media, involving a combination of Darcy's law and percolation models accounting for the disordered nature of the fluid pathways through the rocks^9^. These approaches fail to account for the nanoscale porosity of the kerogen pockets, which leads to strong adsorption effects and an unavoidable breakdown of continuum hydrodynamics as the atom granularity of the fluid becomes non-negligible^10^,^11^. Some corrections have been proposed to account for this breakdown by modifying Darcy's law for slippage through the Klinkenberg effect. While such a formulation accounts for experimental data on gas flow in low-permeability shales^12^, the molecular origin of slippage corrections in this context is not evident owing to the strong attractive molecular interactions between methane and kerogen. Beyond such pitfalls, the dispersed texture of kerogen within the mineral matrix raises the question of the unexplored role of interfacial and wettability effects at their boundaries on hydrocarbon desorption and long-time recovery. One may anticipate that this question is also relevant to a much broader range of situations involving interface-dominated multiphase flow across nanoporous materials, as is ubiquitous in catalysis, adsorption, membrane technology and electrochemistry, for example, supercapacitors^13^,^14^,^15^,^16^.

In this article, we tackle this question by coming back to the microscopic mechanism at stake and climb up the scales from the nanoporous kerogen to the production level. We accordingly address the problem of desorption at wet heterogeneous surfaces and long-time hydrocarbon kinetics at two levels. First, we explore hydrocarbon desorption from a nanoporous membrane mimicking kerogen. Using advanced molecular simulation techniques, we show that, in the presence of the pressure-transmitting (fracking) water, methane desorption is an activated process dominated by interfacial effects, with a wettability-dependent free-energy barrier. In a second step, we demonstrate that such an activated desorption from the nanoporous reservoirs deeply affects the long-time recovery of the hydrocarbons. As a practical implication of the present results, we show that such a multiscale approach involving retarded interfacial transport allows us to explain the unexpectedly fast decline and variable production rates observed in shale gas wells.

## Results

### Activated interfacial transport

We have considered several models of kerogen, accounting for its main features, that is, a porous carbon material with nanometric pores^17^: a disordered hydrophobic nanoporous kerogen, an ordered carbon material, as well as a composite system capturing the hydrophobic/hydrophilic interface associated with shale (Fig. 2a and Supplementary Figs 1 and 2). In the following we will focus on the ordered system, consisting of a hydrophobic nanomembrane represented here as an array of carbon nanotubes (CNTs) of radius *r*. The CNTs are arranged in a triangular lattice, with the void between tubes capped at both ends by a graphene sheet. Despite its simplicity, this robust model captures the main physical ingredients at play in hydrocarbon desorption from nanoporous kerogen through its wet external interface towards the fracture network, while allowing for a systematic variation of the geometrical parameters of the porosity. This is key to gaining fundamental understanding of the mechanism at play. Kerogens are hydrophobic materials with oxygen-to-carbon ratio from a few % up to ∼10%, therefore making our approximation of a pure carbonaceous phase relevant (molecular simulations have confirmed the hydrophobicity of such carbon-rich phases, including the specific case of the disordered matrix considered in this work^18^). As a result, while the exact chemistry will slightly affect adsorption energies, it will not modify the activated mechanism observed in the present work. As for the nanopore size considered in our work, it is consistent with available experimental data that provide evidence for kerogen's significant nanoporosity. Indeed, while several adsorption-based techniques are available to finely characterize the porosity in kerogens, they all lead to pore-size distributions with significant nanoporosity^17^,^19^,^20^ (such nanoporosity has been also evidenced from small-angle neutron scattering^17^). We emphasize furthermore that we confirmed that all different models, ordered or disordered, lead to similar conclusions.

The left side of the membrane is in contact with a reservoir of methane held at constant pressure (*P*_↑_=25 MPa) through the use of a piston. The external surface on the right side of the nanomembrane is covered by a thick film of liquid water, which is left after fracking. The pressure of this fracking water is maintained constant at a pressure *P*_↓_ through the use of a second piston, initially set to 25 MPa before decreasing its value to trigger desorption. While further work is needed to fully characterize the distribution of kerogen in gas shales along with its connections with cracks and fractures, we believe that our model provides a simple yet representative picture of kerogen's nanoporosity and its interface with the external surface. In particular, the use of a wet kerogen interface in our model can be justified as follows. First, considering that kerogen is embedded within hydrophilic minerals such as clay, quartz, pyrite and so on, the most stable configuration corresponds to water adsorbed at this interface while the gas/oil remains trapped in kerogen (this is established in the present paper by means of free-energy calculations for such composite systems, which lead to even larger activation energies). Even for pure kerogen interfaces, the free-energy calculations below show that the stable configuration corresponds to water adsorbed at this interface while methane remains trapped in kerogen's nanoporosity through strong adsorption/confinement effects. Second, even if many kerogen pockets are not in contact with water and therefore empty rapidly on pressure drop, the long-time recovery behaviour will be driven by activated interfacial transport of gas at wet kerogen pockets in contact with water located in the fracture network. As discussed at the end of this paper, the fact that activated interfacial transport potentially describes large-scale observations further supports a model of wet kerogen external surfaces.

We first investigated methane desorption in this molecular model using molecular dynamics simulations, as well as free-energy calculations performed using the umbrella-sampling formalism. Details regarding the models and simulations can be found in the Methods section. Methane desorption from the nanoporous membrane depicted in Fig. 2b was investigated under temperature and pressure relevant to shale reservoir conditions (*T*=423 K and *P*∼25 MPa). The inset to Fig. 2c shows the amount *n*_ex_ of methane extracted from the pores as a function of time *t* for different, yet equivalent, starting configurations; *t* is the time after inducing a pressure drop by decreasing the pressure *P*_↓_ on the right-hand side of the membrane. Despite the pressure difference Δ*P*=−15 MPa imposed across the nanoporous medium, methane remains trapped for long times until it gets extracted while water desorbs from the external surface, with considerable variation in the time before the onset of extraction. This is a typical signature of an activated process. As shown in Fig. 2c, the average timescale *τ*_act_ required to observe methane desorption in the presence of the liquid film at the external surface decreases exponentially with the pressure difference Δ*P<*0:


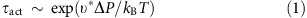


with *υ* a molecular volume; under the conditions of Fig. 2, *υ*=1.2 nm^3^. Such a scaling indicates that fluid desorption through an external surface covered by another (immiscible) fluid is an activated process, possibly inducing important retardation effects in recovery. Counterintuitively, despite such an activated desorption mechanism, fluid extraction occurs at pressure differences Δ*P*, which are still much lower than the Laplace pressure needed to form an oil (methane) hemispherical bubble at the pore mouth (radius *r*) into the external water film: *P*_L_=*γ*_OW_/*r*. For the conditions considered in Fig. 2c, *P*_L_∼100 MPa; this is well above the observed extraction pressures, in the range of 10–20 MPa. This indicates that extraction is actually promoted by thermal fluctuations, which are relevant here due to the nanoscale dimensions of the porous matrix.

To probe the origin of the energy barrier observed in fluid recovery through a wet external surface, we combined molecular dynamics simulations with free-energy calculations in the framework of the umbrella-sampling technique described in detail in the Methods section. Figure 3a shows the free energy Δ*G*/*k*_B_*T* as a function of the amount *n*_ex_ of extracted methane (per unit surface) for different pressure differences Δ*P*. When Δ*P*=0, the stable state corresponds to methane remaining trapped in the porous membrane, with a minute amount of methane solubilized in the adsorbed water film (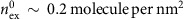). In contrast, large *n*_ex_, which correspond to situations where methane desorbs from the porous membrane, are not favourable, and the corresponding free energy increases beyond 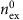 and then plateaus at *n*_ex_=6 molecules per nm^2^. As expected from the data in Fig. 2c, the free energy for Δ*P*<0 exhibits a maximum, although the extracted state is thermodynamically favourable. At large *n*_ex_ the free energy decreases linearly with *n*_ex_, approximately according to d*G*/d*n*_ex_=−*k*_B_*T* ln(*f*_↑_/*f*_↓_), where *f*_↓_ and *f*_↑_ are the fugacities of methane on the downstream and upstream sides of the membrane, respectively. The activated behaviour observed in Fig. 2c is robust as it is also observed using a more realistic disordered nanoporous membrane, which captures the morphological and topological pore disorder in kerogen (sketched in Fig. 2a (II), results in Supplementary Fig. 1). Such a behaviour was also found for a composite hydrophobic/hydrophilic (carbon/silica) membrane, which corresponds to a simple yet physical description of chemical heterogeneities in gas shales (sketched in Fig. 2a (III), results in Supplementary Fig. 2). In particular, while we found that the free-energy barrier increases when more hydrophilic surfaces are considered, it is drastically decreased on applying a pressure drop Δ*P*. This implies that activated transport of hydrocarbon across wet external surfaces remains relevant even when more complex models of gas shales are considered.

Free-energy calculations for different nanotube radii *r* and spacings *D* demonstrate that the free-energy barrier Δ*G* scales with the fraction of the surface occupied by the external surface area, 1−*φ*, where *φ* is the membrane porosity (Fig. 3b and Supplementary Fig. 3). This result suggests that the free-energy difference corresponds to the interfacial free-energy cost of replacing the membrane–water (MW) interface (state I in Fig. 3c) by membrane–methane (MA) and methane–water (AW) interfaces (state II in Fig. 3c). The corresponding surface contribution to the free-energy barrier is


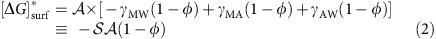


where is the cross-sectional area of the membrane and *γ*_*ij*_ is the surface tension of the interface between *i* and *j* (*i*,*j*=methane (A); membrane (M); or water (W)); and =*γ*_MW_−*γ*_MA_−*γ*_AW_ is the spreading parameter of a methane bubble formed in water at the wet external membrane surface. The trapped state should be favoured when <0 (ref. 20). For non-vanishing pressure drops, one expects a supplementary term *υ*Δ*P* to add to 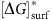
—with *υ*=*l* × *A* a molecular volume corresponding to a wetting molecular film, in line with the previous findings from equation (1). The prediction in equation (2) is found to be in good qualitative and quantitative agreement with the molecular dynamics results in Fig. 3b. Indeed, we performed independent molecular simulations to estimate the surface tensions of the three interfaces using molecular dynamics simulations described in the Methods section. These calculations lead to *γ*_MA_∼16 mJ m^−2^, *γ*_MW_∼82 mJ m^−2^ and *γ*_AW_∼116 mJ m^−2^, and therefore a spreading parameter ≈−18 mJ m^−2^, which is in good agreement with the value estimated from the linear fit in Fig. 3b, =−16.6 mJ m^−2^. The fact that <0 indicates that the confined state, that is, when methane is trapped in kerogen with a water film adsorbed at kerogen's external surface, is thermodynamically stable.

The linear dependence of Δ*G* with the lateral area of the membrane surface 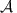 points to the fact that the critical nucleus corresponding to the transition state, as shown in Fig. 3a, extends laterally beyond the maximum lateral size of the simulation box. Therefore, to apprehend the detailed activation process, we extended our investigation using a mesoscale thermodynamic description on the basis of the ingredients identified in the previous molecular approach. Our model considers the free-energy cost to create a methane bubble on the wet heterogeneous membrane. As in the classical nucleation theory, the nucleus shape is obtained by minimizing the surface energy at fixed volume. To account for the full complexity of the heterogeneous nanoporous surface, we performed calculations using the Surface Evolver programme^22^ (details in the Methods section), which we compare with analytical estimates. In these calculations, we used the various surface tensions determined previously using molecular dynamics simulations. Figure 4a shows the free energy Δ*G* of the methane nucleus as a function of its volume *V*_act_ under various conditions, in terms of pressure differences Δ*P* and pore geometry. Here we normalized the free energy and the volume by characteristic quantities 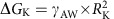 and 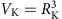, with the Kelvin radius *R*_K_=*γ*_AW_|Δ*P*|.

For each volume *V*_act_ and pressure difference Δ*P*, the solution of the free-energy minimization corresponds to a nearly spherical methane cap having a contact angle *θ*_eff_ (Fig. 4b). Interestingly, *θ*_eff_ is very close to the solution of the Cassie–Baxter equation, which describes the effective contact angle *θ*_eff_ on the porous surface as a linear combination of the contact angles on the solid (*θ*_solid_=32°) and on the porous domains (*θ*_pore_=0°):


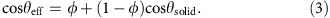


It is interesting to note that the notion of Cassie–Baxter composite wetting extends here to the description of free-energy barriers and transition states on heterogeneous surfaces.

Using the spherical cap approximation, a straightforward calculation shows that the corresponding free energy of the spherical cap is given by


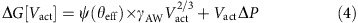


where 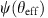 is a geometrical term that depends only on the effective contact angle *θ*_eff_, defined by the expression given in the Methods section. In the limit of small contact angle *θ*_eff_, one has 
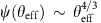
. We show in Fig. 4a that the free energy predicted using the spherical cap approximation, with the values of the effective contact angle *θ*_eff_ obtained by a fit to the Surface Evolver results using equation (4), are in very good agreement with the complete numerical calculations of the nucleus shape. The fitted contact angles are within 2° of those predicted by the Cassie–Baxter equation (values in Supplementary Table 1), the slight difference resulting from spatial distortions induced by the line tension contribution exerted at the contact line between the three coexisting phases (kerogen, methane and water). The free-energy barrier for methane desorption is then obtained by maximizing Δ*G*[*V*_act_], leading to 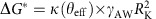 with *R*_K_=γ_AW_/|Δ*P*|; the geometrical term, given in the Methods section, takes the form 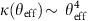 in the limit of small *θ*_eff_.

### Long-time kinetics of methane recovery

**Figure 1:**
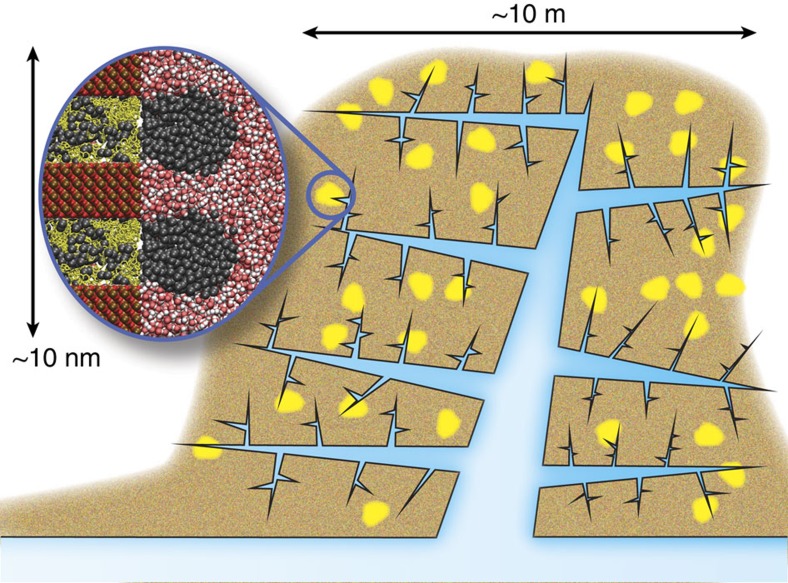
Hydrocarbon recovery from unconventional reservoirs. Schematic illustration of a fracture network (blue), created by hydrofracking, penetrating previously isolated hydrocarbon-rich kerogen pockets (yellow) within a mineral matrix (brown). Here we consider the post-fracking situation in which water within the hydrofracking network is in contact with the kerogen surface. Extraction of the hydrocarbon requires formation of a nucleus with a high interfacial energy. The zoomed image illustrates such a scenario, in which a methane nucleus (dark grey) forms at a kerogen surface (yellow) adjacent to hydrophilic mineral surfaces present in shales (here quartz, with Si and O atoms as red and golden spheres). Considering other inorganic phases such as clays will lead to the same consistent picture of interfacial activated transport as they have similar wetting properties towards methane and water. However, local variations in surface chemistry and geometry will determine the magnitudes of the energy barriers preventing extraction, which will have a broad range of values due to the heterogeneous, multiscale texture of the shale.

Altogether, the microscopic and mesoscale approaches above point to activated desorption at heterogeneous interfaces, and allow quantitative estimates for the free-energy barrier for hydrocarbon extraction from nanoporous media. These physical ingredients are expected to deeply impact the dynamics at large scales, but they have not been included up to now in the description of hydrocarbon recovery. Several key features emerge from the above description that allow the identification of crucial limiting steps in hydrocarbon extraction. First, the possible range of energy barriers, which depend on the porosity and pressure difference, is found to be of about a few tens of *k*_B_*T* for standard recovery conditions Δ*P*∼−15 MPa. In the framework of the nucleation theory, the activation time is given by an Arrhenius law *τ*_act_=*τ*_0_ exp(Δ*G*/*k*_B_*T*) with *τ*_0_∼10^−13^ to 10^−12^ s a typical microscopic attempt time. This leads to timescales *τ*_act_ of the order of a month to years, which are relevant to the typical production declines observed in shale gas recovery^3^. A second important insight from the approach above is the strong dependence of the energy barrier on the effective contact angle, which scales as 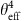 for small *θ*_eff_. Returning to the picture of a collection of kerogen pockets dispersed in a mineral matrix (Fig. 1), one expects, therefore, a broad distribution of effective contact angles for the various individual reservoirs, due to wetting and geometrical variability. In turn, this induces an even broader distribution of free-energy barriers ΔG* because of the scaling relation 
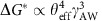
. Such a variability in the free-energy barriers, which are expected to be drastically affected by the local physical chemistry of the kerogen and the presence of surfactants, leads to activation times *τ*_act_ that are also widely distributed. Although in a different context, this picture shares ingredients with the long-time kinetics for capillary condensation in granular materials, leading to logarithmic ageing^23^. Typically, for a given time *t*, only the reservoirs with an activation time *τ*_act_ smaller than *t* have desorbed. The recovered amount is calculated in terms of the number of active reservoirs. The overall gas volume (*t*) extracted at a time *t* is accordingly


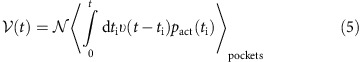


where 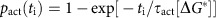 is the probability of a pocket with a particular energy barrier being overcome at a specific time; A is the total number of gas pockets; and 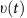 is the (time-dependent) volume of extracted gas once a barrier has been overcome. The latter increases from zero to a maximum of, say, *V*_0_ over a time *τ*_h_, which is the typical time to empty a single reservoir.

To estimate (*t*), a specific distribution of the effective contact angle and surface tension should be used to estimate the distribution of energy barriers Δ*G*. The crucial point is, however, the broad variability of these parameters among reservoirs (for a given Δ*P*). To simplify the analysis and obtain analytical predictions, we assume that they follow a simple exponential distribution but the precise form is not critical as discussed below. Using 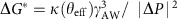, the energy barrier distribution is accordingly in the form


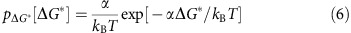


with 
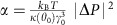
; *θ*_0_ and *γ*_0_ fix the typical range spanned by these parameters over the reservoirs. While such an exponential distribution represents merely one possible form to estimate (*t*), the specific distribution considered for Δ*G* is not crucial; the key ingredient in our model to predict the short-time and long-time algebraic decays is the existence of an energy barrier, taking values over a broad interval. Such an energy barrier introduces a typical activation time *τ*_act_, which defines a short-time *t*<*τ*_act_ and a long-time *t*>*τ*_act_ regimes.

We solve this problem for two regimes that depend on the time *τ*_h_ to empty a single reservoir (details of the steps involved are provided in the Methods section). For *t*<*τ*_h_, we must consider the dynamical process during this emptying, and one expects 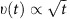 as predicted from a classical boundary-limited flow applied to a single pocket using Darcy transport leading to diffusion-like equation (5). We predict that the rate of recovery will then scale as 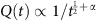. In the long-time regime *t*>*τ*_h_, the finite emptying time can be neglected and the need to overcome ever-larger energy barriers then limits the rate of recovery. Accordingly, we predict


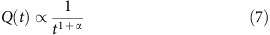


where 
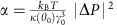
 is a non-universal exponent, which strongly depends on thermodynamic conditions (*T*, Δ*P*) but also on the specific interactions with the gas shale components through upper limits *θ*_0_ and *γ*_0_ on the local effective contact angle and surface tension. Beyond the typical time *τ*_h_ needed to empty a single reservoir, the number of active reservoirs decays rapidly as the energy barrier that must be overcome to activate them becomes unreachable. The statistical model above shows that the activated kinetics of hydrocarbon recovery departs from the overall classical boundary-limited flow, which predicts *Q*(*t*)∼*t*^−1/2^ for a single reservoir. As shown by equation (7), the recovery is predicted to exhibit a faster decline for long times, with an exponent of the algebraic decline of the order of unity, although dependent on the pressure protocol used to trigger recovery and on the local characteristics of the well under investigation.

## Discussion

The statistical model developed in our paper predicts a two-regime scenario for the production decline with strong dependence on the fracking fluid through its wetting properties and miscibility in hydrocarbon. Rigorous validation of this critical prediction against field-scale data requires more work, including experimental investigation on simple systems before moving to gas shale. Moreover, shale production data display a wide variety of length and timescales associated with complex phenomena (geomechanics, transport and so on), which makes direct comparison with our model premature unless intermediate validation steps are added. Nevertheless, at this stage, it is important to check that our prediction, that is, activated transport across wet kerogen interfaces, is compatible with real data. The Supplementary Discussion section contains a discussion of a large collection of field scale data on gas production over time for some typical examples of unconventional wells from different shale plays. The statistical model presented in our paper is consistent with the general experimental behaviour; the two-step algebraic decline predicted by our statistical model, with a more rapid decline at long times than at short times, is compatible with short- and long-time extraction rates previously identified in field scale data. Moreover, by including ingredients such as interfacial and physical chemistry effects, such a multiscale model also accounts qualitatively for the effect of changing the fracking fluids (non-water fracking fluids tend to have smaller decline exponents).

**Figure 2:**
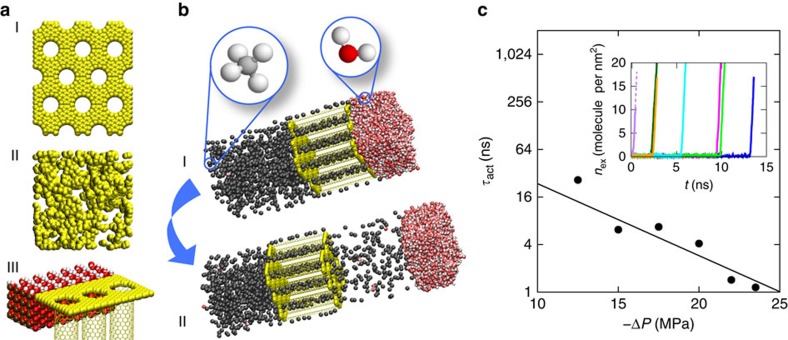
Interfacial transport in nanoporous media under applied pressure difference. (**a**) Three model kerogens were investigated: (I) an ordered CNT array; (II) a disordered nanoporous carbon material; and (III) a composite membrane containing hydrophilic (quartz) and hydrophobic (CNTs) regions. (**b**) Methane (dark grey) is initially confined within a CNT membrane (yellow) arranged in a triangular lattice. (I) The left side of the membrane is in contact with a reservoir of methane held at constant pressure *P*_↑_=25 MPa through the use of a piston (not drawn). The right side of the membrane is covered by a thick film of liquid water (red and white) with a pressure maintained constant at *P*_↓_ through the use of a second solid piston (not drawn). At a time *t*=0, a pressure difference Δ*P*<0 is applied to extract methane by decreasing *P*_↓_ (II). Different nanotube radii *r* and pore spacings *D* were considered (here *r*=0.59 nm and *D*=1.70 nm). (**c**) Average time *τ*_act_ until an escape event as a function of the applied pressure difference Δ*P*, with the solid line indicating an exponential fit to the points (*τ*_act_∼exp(*a*Δ*P*) with *a*=0.21±0.04 MPa^−1^). The insert shows different equivalent simulations used to estimate *τ*_act_; under exactly identical temperature and pressure conditions (here Δ*P*=−15 MPa and *T*=423 K) but different (here 7) initial configurations, the amount, *n*_ex_, of methane extracted from the membrane per unit of surface area is monitored as a function of time *t*.

The field-scale data gathered in Supplementary Fig. 4 also indicate that in general the presence of two regimes is clearer in hydrofracked wells compared with those stimulated with other fracking fluids containing little or no water. Such a dependence on the fracking fluid is consistent with the activated recovery found in the present work; indeed, while hydrofracking requires the system to overcome energy barriers to initiate hydrocarbon extraction, such a behaviour is not expected for fluids that are miscible with the hydrocarbon fluid such as liquid petroleum gas and CO_2_ in specific temperature and pressure ranges. To validate this conjecture, we carried out a series of additional simulations in which water was replaced with CO_2_. After an equilibration stage, during which an additional force field prevents methane from leaving the pores and CO_2_ from entering, a pressure gradient is applied and the system is monitored over time. Multiple repeats were performed using systems with equivalent initial states to test for the presence of an energy barrier. These results, plotted in Fig. 5, show that unlike the water simulations (Fig. 2c), no retardation in the transport of methane out of the membrane was observed, therefore suggesting that no energy barrier exists to inhibit extraction in this case. Furthermore, CO_2_ reliably replaces methane within the pores, as shown by the blue markers in Fig. 5. This presents a win-win strategy in which CO_2_ as a fracking fluid reduces the environmental impact of the process while allowing efficient CO_2_ capture within the shale reservoir at the end of the process. While CO_2_ is already used for conventional reservoirs in the framework of enhanced oil recovery, limitations of CO_2_ as a fracking fluid have been identified such as its low viscosity, high compressibility and poor proppant carrier properties. However, the role of CO_2_ proposed here, as an alternative to hydrofracking, is fundamentally different^24^; CO_2_-fracking eliminates activated interfacial transport at the external surface of kerogen. By replacing water with propane, we also observed that this alternative fracking fluid leads to non-activated interfacial transport owing to its favourable interactions with the confined hydrocarbon (results not shown). However, while CO_2_ replaces methane in kerogen, propane was found to be recovered together with the hydrocarbon phase on extraction. This suggests that different recovery strategies, that is, allowing CO_2_ capture or efficient energy extraction without hydrocarbon loss, can be envisaged by playing with the different surface interactions at play through the choice of the fracking fluid. Despite the benefits of using fracking fluids that eliminate activated transport on shale gas extraction, further investigation is required to include possible swelling effects as CO_2_-fracking, for instance, is known to swell kerogen and reduce shale permeability.

Our novel framework emphasizes that new paradigms must be envisioned to understand hydrocarbon extraction from unconventional reservoirs. These new insights into transport at the nanoscale suggest new leads for the industry and pave the way for the rational adjustment or re-design of existing processes to minimize the retardation due to these interfacial effects. In shale gas extraction, control can be obtained over the surface tensions, and therefore the energy barriers, by altering the composition of the pressure-transmitting fracking fluids.

Beyond shale gas, we expect that such activated desorption phenomena will be of paramount importance for any field involving nanoporous media in which nanoscale fluid interfaces are present. While structural defects at the external surface of nanoporous materials have been identified as limiting steps in transport and reactivity in confined geometries^16^,^25^,^26^,^27^, retardation effects arising from the extraction of a liquid phase into an immiscible liquid wetting the external surface are unprecedented. Such effects are related to, but distinct from, fluid–fluid and fluid–solid interfaces, which are known to resist or drive transport in nanopores via the Laplace pressure^28^,^29^. Manipulation of interfacial parameters in such systems allow envisioning rational control over transport inhibition in a variety of contexts such as membranes, catalysis and chromatography.

## Methods

### Simulation model

We used the open-source LAMMPS software to carry out molecular dynamics simulations, employing a velocity-Verlet algorithm with a timestep of 2 fs. Figure 2b illustrates the geometry of the simulations containing a porous membrane. We define the coordinate system such that the axis of the pores was aligned with the *z* axis, with the methane reservoir at the more negative end. The membrane pores are composed of CNTs with a zig-zag configuration, arranged into a triangular lattice with the rows of the lattice aligned in the *x* direction. Graphene sheets at the ends of the pores block the voids between the CNTs, with the centre of each nanotube aligned with the centre of an graphene ring. The nanotubes ends are cleared by removing atoms in the graphene sheet located within *r*+1.42 Å of the CNT centre, the extra 1.42 Å equal to the carbon–carbon bond length, ensuring a reasonable spacing between carbon atoms. We apply periodic boundary conditions in the *x* and *y* directions, and ‘shrink-wrapped' boundary conditions in the non-periodic *z* direction, allowing the system to expand and contract as necessary. The initial state is created by running the simulation at 25 MPa for 1 ns while preventing the movement of methane out or water into the pores using an additional repulsive force field at the pore opening.

We use a Lennard–Jones potential to model the non-electrostatic forces between all particles, with the form


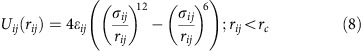


where *r*_*ij*_ is the separation between two particles and *r*_c_=13.5 Å is the force cutoff distance for interactions not involving piston atoms (which we describe later). The values of the *σ*_*ij*_ and *ɛ*_*ij*_ parameters for different types of interacting particles are summarized for particles of the same type in Supplementary Table 2. For interactions between unlike particles, we use Lorentz–Berthelot combining rules, such that for particles of type *α* and *β*, 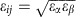 and 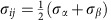. The exception to these combining rules is the methane–piston interaction, which we describe below.

We use a united atom description of methane with the TraPPE force field^30^. Each methane molecule is represented by a single Lennard–Jones particle, with interaction parameters as listed in Supplementary Table 2. This model accurately describes the liquid–vapour coexistence curve and critical temperature of methane. We include at least 40 methane molecules per square nanometre of membrane cross-sectional area.

We employ the simple point charge (SPC) water model^31^, with the charges and Lennard–Jones parameters given in Supplementary Table 2. The bond angle is fixed at 109.47° and the hydrogen–oxygen bond length at 1 Å using the SHAKE algorithm^32^. For the electrostatic interactions, we truncate the force at 9 Å. Owing to the lack of periodicity in the *z* dimension, Ewald sum methods cannot be used for the long-range electrostatic forces. While the use of a cutoff affects the value of the surface tensions, this simplified water molecule still served the purpose of providing a polar fluid, which is immiscible with methane. We included at least 100 water molecules per square nanometre of membrane lateral area.

For simulations involving CO_2_, we use the ‘elementary physical mode' described by Harris and Yung^33^, with interatomic interaction parameters as described in Supplementary Table 2. The carbon–oxygen bond length is fixed at 1.149 Å using the SHAKE algorithm, while constraining the bond angle *θ* by a harmonic potential 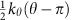 with *k*_*θ*_=1,236 kJ mol^−1^ rad^−2^.

A Langevin thermostat acting on the methane and oxygen atoms with a friction coefficient of 0.01 fs^−1^ holds the system temperature at 423 K. The thermostat acts only in the *x*–*y* plane so as not to interfere with the transport of the fluid along the axis of the pores.

Opposing pistons with a graphene-like structure apply a prescribed pressure to each side of the membrane. Piston atoms are constrained such that they only move along the *z* axis. At each time step, the force on each piston atom is set to the average force of all atoms in the piston—causing them to move in unison—plus an additional component corresponding to the external pressure on that piston. We define the interaction between piston and fluid atoms by a Lennard–Jones potential truncated at the minimum energy (*r*_*c*_=2^1/6^*σ*_*ij*_), and therefore purely repulsive. To prevent methane accumulation at the downstream piston (in contact with the water phase), we use a large *σ*_*ij*_ value of 6.8 Å for methane–piston interactions.

### Activation time determination

In the simulations used to generate the data shown in Fig. 2c we used a membrane with a pore radius *r*=0.59 nm and pore spacing *D*=1.70 nm, and simulation dimensions in the periodic dimensions *x* and *y* of 1.704 and 2.951 nm, respectively, such that the system contained two nanotubes. After the initialization process described above, we equilibrated the system with no pressure difference for 2 ns, then linearly decreased the pressure on the left (positive *z*) side of the membrane containing the water over 10 ps. The simulation was run until escape was observed (and for a short time after). We report results for six pressure differences, for each of which we simulated seven trials with different but equivalent initial conditions. The activation times for the individual trials are shown in Supplementary Fig. 5.

### Umbrella-sampling molecular dynamics simulations

We used umbrella sampling to measure the free energy as a function of the amount of extracted methane and gain insight into the origin of the energy barrier. Umbrella sampling is a method commonly used to study the thermodynamics of rare events inhibited by energy barriers^34^. An order parameter must first be identified describing the process of interest. We used the number of extracted methane molecules per unit area as an order parameter, *n*_ex_, which we have approximated using a sigmoidal function to make the variable continuous and differentiable:


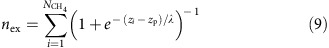


where *z*_*i*_ is the *z* coordinate of the *i*th methane particle, *z*_p_=1.8 Å relative to the centres of the carbon atoms in the external graphene surface and *λ*=0.25 Å. We associate a biasing potential *ω*(*n*_ex_) with the order parameter to force the system into an ordinarily unlikely state. By measuring the probability distribution of *n*_ex_ in the biased simulation, *P*^B^(*n*_ex_), we deduce the unbiased free energy from the relationship


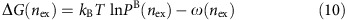


In practice, rather than attempt to find a bias potential, which allows the entire domain of *n*_ex_ to be sampled, it is more practical to run many simulation ‘windows'. In each window we applied a simple harmonic bias potential so as to sample a particular range of *n*_ex_, with the form


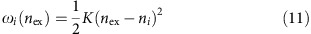


where *K* and *n*_*i*_ determine the strength and centre of the bias for the *i*th window. To implement such a bias in a molecular dynamics simulation, the force on each methane particle resulting from the bias must be described as a function of the particle position along the *z* axis:


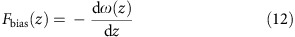



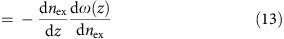



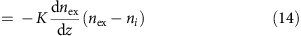


The need for *n*_ex_ to be differentiable with respect to the particle position *z* motivates the use of the logistic form in equation (9), for which the derivative is well known and straightforward to implement:


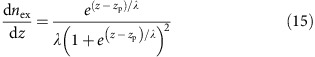


To calculate the full unbiased probability distribution *P*^U^, we used a weighted average of the unbiased probabilities in each window, 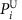, according to the weighted histogram analysis method^34^. The weightings were calculated so as to minimize the statistical error in *P*^U^:


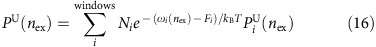


where 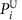 and *N*_*i*_ are the measured unbiased probability and number of samples, respectively, in the *i*th window. The *F*_*i*_ terms were calculated according to


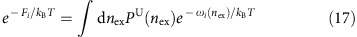


We found a self-consistent solution by starting from equation (16) with *F*_*i*_=0 for all windows, then iterating between equation (17) and (16) until convergence^34^. We considered the solution to have converged when the maximum value of (1−*F*_old_/*F*_*i*_)^2^ for any window was <10^−15^, where *F*_old_ is the value of *F*_*i*_ in the previous iteration.

After unblocking the pores and applying the bias, we equilibrated the initial simulation window at *n*_*i*_=0 for at least 1 ns. To measure *P*^B^(*n*_ex_), we sampled the system every 0.2 ps for at least 1 ns. We generated additional windows by restarting a simulation equilibrated with a similar *n*_ex_, equilibrating for at least 0.2 ns. We typically simulated windows separated in *n*_*i*_ by 0.4 molecule per nm^2^, though when close to a free-energy maximum we sometimes required additional windows separated by 0.1 molecule per nm^2^, and with larger spring constant. We list the membrane parameters for the simulations used to produce the results in Fig. 3a in Supplementary Table 3 (system a).

### Disordered membrane

To test the generality of the effects observed for the model CNT membrane, we carried out umbrella-sampling simulations in a system with a 5 × 5 × 5 nm disordered porous carbon membrane obtained using an atom-scale reconstruction technique. The pore size, chemical composition (including *sp*^2^/*sp*^3^ hybridization), and morphological disorder of this structure are comparable with those of kerogen^35^,^36^,^37^,^38^.

### Heterogeneous membrane

In complex porous materials such as gas shale, a wide variety of geometries and surface chemistries may be present. We have explored the impact of such heterogeneities using a model composite membrane consisting of nanoporous hydrophobic component of width 3.12 nm, similar to the ordered hydrophobic membrane already described, and a hydrophilic component represented by a quartz surface of width 2.16 nm. This membrane is pictured in Fig. 2a(III) and the upper-left inset in Supplementary Fig. 2. The length of the simulation domain in the direction parallel to the stripes was 5.106 nm. The quartz surface was prepared by cutting a bulk quartz lattice^39^ along the (100) plane, then attaching protons to the resulting dangling oxygen bonds and allowing them to relax over a short NVE simulation. The quartz atoms are frozen during the simulations. The charge and Lennard–Jones parameters for the silicon, oxygen and hydrogen from which the quartz is composed are listed in Supplementary Table 4. The water was completely wetting on the quartz, while showing a contact angle of 133° on the graphene component (see Supplementary Fig. 6 and below in the Methods section), higher than typically observed for graphene because only a single layer was used. The free energy as a function of extracted methane for Δ*P*=0 and two additional pressure differences as measured using umbrella sampling is shown in Supplementary Fig. 2.

### Free-energy barrier dependence on porosity

**Figure 3:**
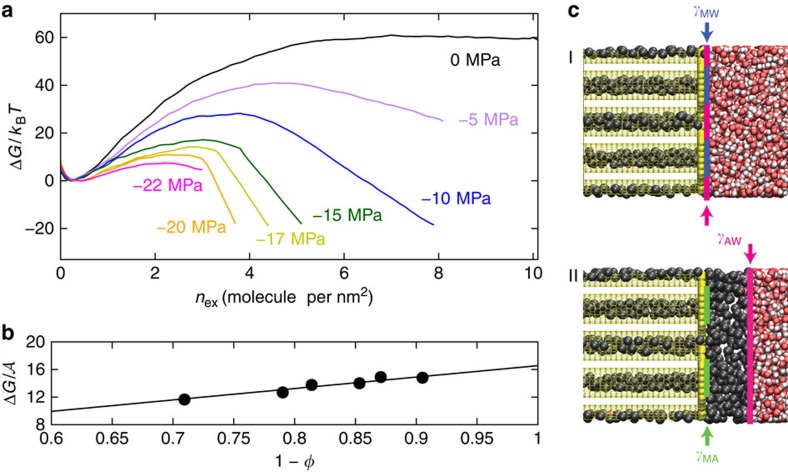
Activated desorption across wet external surface. (**a**) Free energy Δ*G*/*k*_B_*T* calculated using umbrella sampling as a function of extracted methane per unit area, *n*_ex_, for several pressure differences Δ*P* (indicated in the graph). The free energy is given relative to the local minimum at low *n*_ex_. The pore radius is *r*=0.59 nm and pore spacing *D*=1.7 nm. (**b**) Change in free energy per unit area Δ*G*/A (in mJ m^−2^ ) between the confined (I) and extracted (II) states when Δ*P*=0 as a function of the solid fraction of the surface, 1−*φ*. The pore radius *r* and spacing *D* of each point are listed in Supplementary Table 3 (systems a and d–h in the table). The straight line indicates a fit to Δ*G*/A=−(1−*φ*) with **=−16.6±0.4 mJ m^−2^. (**c**) Typical molecular configurations corresponding to the confined (I, low *n*_ex_) and extracted (II, high *n*_ex_) states. The dark grey spheres are for methane molecules while the red and white spheres correspond to water molecules (the nanoporous membrane is shown in yellow). For each system, we also show the different interfaces: MW; AW; and MA.

To test the linear scaling with 1−*φ*, we used umbrella sampling to determine the free energy as a function of *n*_ex_ for different combinations of pore diameter and spacing listed in Supplementary Table 3 (systems a, d–h). To calculate the porosity, a correction term *c* was added to the pore radius to correct for the finite size of the carbon atoms, such that 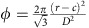. The results confirm the linear relationship in equation (2), as seen in Fig. 3, with the best fit found for *S*=16.55±0.41 mJ m^−2^ and *c*=0.10±0.03 nm.

### Surface tension calculation

To verify the proportionality with the spreading parameter in equation (2), we measured the surface tensions of the three interfaces of interest in a separate set of molecular dynamics simulations. We prepared three systems: one in which a water phase and methane phase are held in contact under pressure from two graphene pistons, much like the simulations already described, but without any membrane separating the phases; another with only a water phase between the pistons; and another with only methane. The systems are 5-nm wide in the *x* and *y* directions, and contain 7,200 water molecules and/or 910 methane molecules. The potentials used to model interactions between the piston atoms and other species are identical to those used for the membrane atoms in umbrella sampling simulations, listed in Supplementary Table 2, with the force truncated at 13.5 Å. We equilibrated the systems for 2 ns before beginning the surface tension measurement. In the methane–water mixed system we initially equilibrated for an additional 1 ns with a planar force separating the phases. We simulated the methane–water systems for 15 ns, water-only for 18.4 ns and methane-only for 10 ns, taking samples of the configuration every 1 ps.

We can express the surface tension between phases A and B using the Kirkwood and Buff approach^40^, as an integral across the interface of the difference between the pressure components normal and tangential to that interface, *p*_N_(*z*) and *p*_T_(*z*):


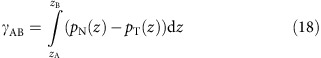


where the *z* axis is normal to the plane containing the interfaces, and *z*_A_ and *z*_B_ correspond to points within the bulk of the A and B phases. The normal and tangential pressures can be written in terms of the pressure tensor elements, *p*_N_=*p*_*zz*_ and 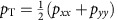.

The pressure tensor components themselves contain a kinetic component, equivalent to that of an ideal gas, plus a second component taking into account the interactions between molecules. The diagonal elements of the pressure tensor at a position *z*, *p*_*αα*_(*z*), where *α*=*x*, *y* or *z*, can be calculated using the expression


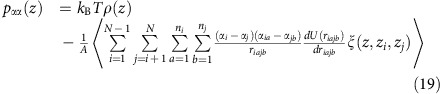


where the sums are over each interatomic interaction between each pair of molecules, each molecule being composed of *n*_*i*_ atoms. *α*_*i*_ and *α*_*ia*_ denote the *α* coordinate of the centre of mass of the *i*th molecule, and the *a*th atom of the *i*th molecule, respectively. *r*_*iajb*_ is the separation between atoms *ia* and *jb*, *ρ*(*z*) is the fluid density and *A* is the simulation cross-sectional area in the *x*–*y* plane. We count the frozen graphene atoms as individual molecules when calculating *p*_*zz*_, but we neglect them in the calculation of *p*_*xx*_ and *p*_*yy*_ due to the symmetry in these dimensions.

The function 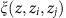 depends on the choice of the contour drawn between particles 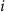 and 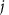 along which the contribution of that interaction is distributed. There is no unique definition for this contour^41^. We use the Irving and Kirkwood convention^42^, which has been shown to agree with other methods^43^. The contour in this model is a straight line connecting the two interacting molecules, such that *ξ*(*z*, *z*_*i*_, *z*_*j*_) takes the form:


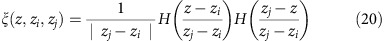


where *H*(*x*) is the Heavyside step function. In practice, we divide the system along the *z* axis into slabs of width 1 Å, and split the contribution to the pressure tensor of each interaction equally between any slabs between or containing the two interacting particles.

At equilibrium, the normal component of the pressure tensor must in principle be constant and equal to the pressure applied by the pistons. In practice the length of time for which the system must be sampled before this limit is reached for the water phase is very long. We have made the assumption that the normal pressure would eventually converge in our calculation of the surface tension.

The tangential and normal pressure profiles calculated using this method are shown in Supplementary Fig. 7. The measurements of *γ*_MA_ and *γ*_MW_ in the mixed system are consistent with the more precise measurements in the single-component systems.

### Surface energy minimization with Surface Evolver

To overcome the limitation imposed by the small molecular dynamics simulation box size and construct a model for the critical nucleus, we have employed a mesoscale thermodynamic approach. We use the energy minimization algorithm implemented in the open-source Surface Evolver programme^22^ to determine the nucleus geometry at a range of volume.

Surface Evolver represents surfaces by mesh of triangular facets. In a standard minimization step (default ‘g' command), the force on each vertex is calculated based on the gradient of the free energy, and then the vertices move according to the strength and direction of that force. Alternatively, the ‘Hessian seek' command allows the Hessian matrix of second derivatives to be used to find the minimum energy configuration in the direction of motion as determined by the forces. These two techniques can be alternated while minimizing the energy. Further details of the minimization algorithm can be found in the Surface Evolver manual.

In the absence of a pressure difference, the total energy of the system can be described by


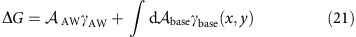


where *γ*_base_(*x*,*y*) is a periodic function of circular domains representing the pores, arranged in a triangular lattice. The function is equal to −*γ*_AW_ within these circular domains, and *γ*_MA_−*γ*_MW_ everywhere else.

The surface tensions calculated using the molecular dynamics simulations described above were used for the minimization calculations.

During the evolution of a surface in Surface Evolver it is usual to start with a crude approximation to the final geometry, then alternating between moving towards the minimum energy, and refining the surface by splitting existing facet in half. We restricted the refinement of the base of the nucleus such that new vertices are only created on the contact line, since additional vertices within the base perimeter are redundant, experiencing no net force.

The initial geometry consists of 50 vertices arranged on the substrate in a circle to form the contact line. Each of these contact line vertices are connected by an edge to their two neighbours, to a vertex in the centre of the circle, and to a vertex positioned above the centre of the circle at the height required for the target nucleus volume. Vertices located on the contact line are constrained such that they only move in the plane of the surface. Four initial contact radii were tested for each volume, and all but the lowest energy result discarded.

Surface Evolver minimizations of drop or bubble geometries on patterned surface can be challenging due to a tendency for the system to become stuck in local minima. If care is not taken a situation can arise in which even the liquid–vapour interface obviously fails to adopt a physically reasonable shape. We have found an effective strategy to avoid such problems is to alternately evolve the surface with the contact line vertices fixed in place or allowed to move freely. We used a combination of the regular linear gradient decent method (the default ‘g' command) and the Hessian seek method (‘Hessian_seek' command), described above. Motion of the vertices in both cases is multiplied by a ‘scale factor' (<1), automatically calculated by the software, which dampens the motion as an energy minimum is approached. If the scale factor approaches zero, the surface stops evolving, while not necessarily having found the minimum energy configuration.

The algorithm described in Supplementary Note 1 was used within Surface Evolver to minimize the interfacial free energy.

### Cassie–Baxter model of the nucleus

**Figure 4:**
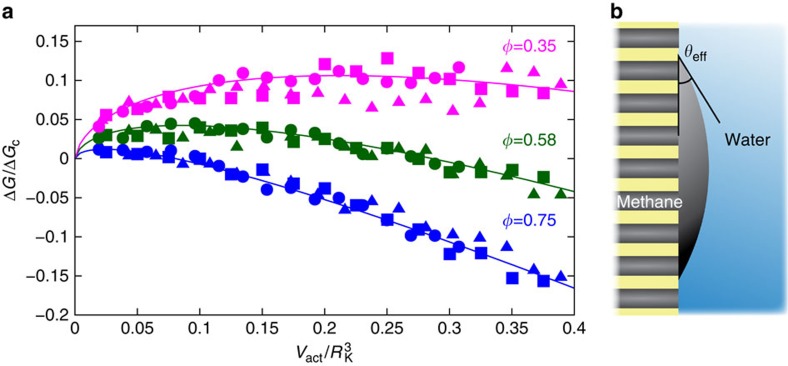
Surface energy minimization and critical nucleus. (**a**) Free energy Δ*G* of the methane nucleus as a function of its volume, *V*_act_, as calculated by surface energy minimization for different pressure differences (10, 12.5 and 15 MPa for circles, squares and triangles, respectively). Different pore surface fractions *φ* (indicated in the graph) are considered. The free energy is normalized by 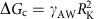 and the volume by 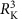, with the Kelvin radius *R*_K_=*γ*_AW_/|Δ*P*|. In this plot, for a given surface geometry—as characterized by the corresponding porosity *φ*—data for various pressures drops collapse onto a single curve. Solid lines show the predicted free energy assuming a spherical cap geometry with an effective contact angle *θ*_eff_ determined by a fit to the Surface Evolver results. For each geometry, the fitted *θ*_eff_ is within 2° of the value predicted using the Cassie–Baxter equation for wetting on heterogeneous surfaces (Supplementary Table 1). (**b**) Schematic representation of the geometry of the contact angle formed by a methane spherical cap (grey phase) at the interface between a nanoporous membrane (yellow) and water (blue). *θ*_eff_ is the contact angle as described in the Cassie–Baxter equation.

**Figure 5:**
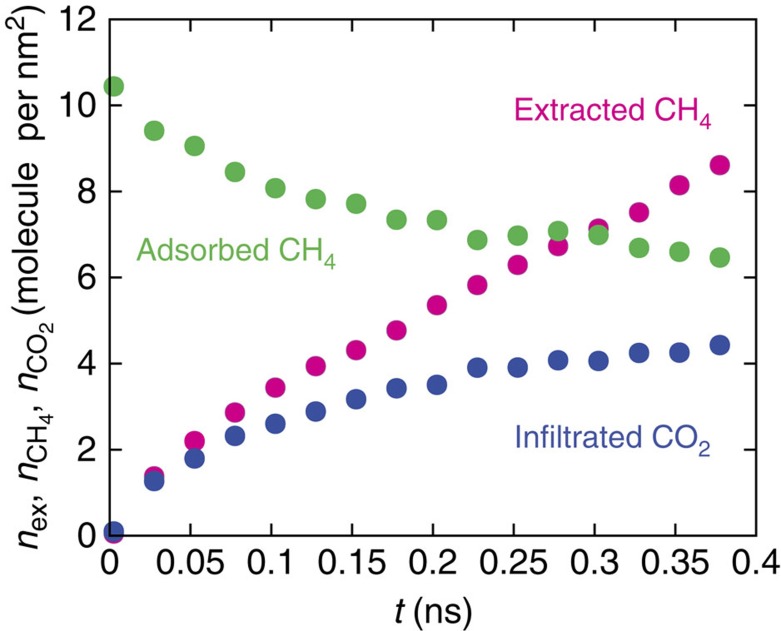
CO_2_ as an alternative fracking phase. The number of methane molecules extracted (magenta), adsorbed in the pores (green), and the number of fracking phase CO_2_ molecules injected into the nanoporous membrane (blue) over 0.2 ns of unbiased molecular dynamics simulation. The pressure difference is Δ*P*=−20 MPa. The symbols correspond to the average values over seven equivalent simulations, that is, ‘repeats', with different initial conditions. When CO_2_ is used as the fracking fluid, methane immediately desorbs from the pores with no evidence of an energy barrier limiting its recovery, in contrast to Fig. 2c.

Here we describe the derivation of the thermodynamic model to predict the size and energy of the critical nucleus, which we compare with the Surface Evolver results in Fig. 4.

Consider a methane nucleus with volume *V*_act_ on a porous surface. We make the simplifying assumption that the nucleus adopts an idealized spherical cap geometry with base radius *R* and an effective contact angle *θ*_eff_. The base radius *R*, contact angle, volume and curved surface area A_AW_ (methane–water interface) are related by


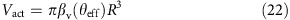



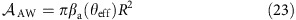


where 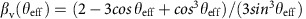 and 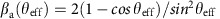. In the limit of low *θ*_eff_, *β*_v_(*θ*_eff_)=*θ*_eff_/4 and *β*_a_=1.

Assuming the length scale of the pattern is significantly smaller than the radius of the nucleus, the total free energy can be written


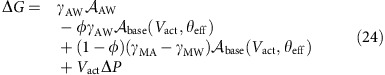


In the case of the simple geometry used in the molecular dynamics and surface evolver calculations, 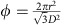. The free energy can be rewritten in terms of the contact angle of the methane on the solid phase:


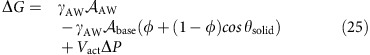


into which we may substitute the expressions for the area, volume and the Cassie–Baxter effective contact angle, to produce an expression for the free energy in terms of *γ*_AW_, *V*_act_, *θ*_eff_ and Δ*P*:


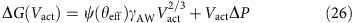


where 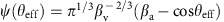. In the limit of low *θ*_eff_, 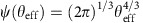. It is straightforward then to determine the maximum in the free energy:


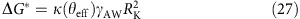


where we introduce the Kelvin radius *R*_K_=γ_AW_/|Δ*P*| and a geometric factor 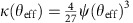. In the small angle limit 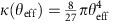.

### Measuring hydrophobicity of the membrane

To confirm that the model membrane is hydrophobic, we carried out a molecular dynamics simulation of a water drop on a graphene substrate. We simulated 700 water molecules represented by the SPC model described above section in an initially cubic lattice. During the initial 0.5 ns, a velocity rescaling thermostat ramped the temperature linearly from 400 to 300 K—a process we find allows the drop to relax more rapidly. The drop momentum in the *xy* plane was reset to zero every 20 ps during this first 0.5 ns. After the initial relaxation, the temperature was held at 300 K using Langevin thermostat. After equilibrating for a further 3.5 ns the system was sampled every 1 fs over a 2-ns period so as to measure the contact angle.

The radial density profile of the drop, measured relative to the centre of mass, is shown in Supplementary Fig. 6. To obtain the position of the surface as a function of *z* and 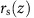, the system is divided into slabs parallel to the substrate and 0.5 Å thick in *z*. In each slab we fit the radially averaged density to the function 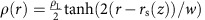 via 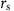, the position of the interface; and 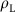, the bulk density, fixing 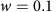 nm. To measure the contact angle, a circle is fitted to *r*_s_(*z*) in the central region of the drop, avoiding deviations due to contact line tension near the base and low density regions near the top.

### Statistical model of long-time recovery

Here we describe with additional detail the derivation of the statistical model for long-time recovery kinetics. We begin by considering the shale as containing a large number of trapped gas pockets, A, each containing a volume of gas *V*_0_, and each of which must overcome some energy barrier ΔG* before the gas within may be recovered. This scenario is illustrated schematically in Fig. 1. These pockets have a wide distribution of energy barriers as a result of variations in the local geometry and surface chemistry of the shale. The variation in energy barriers leads in turn to variations in activation time *τ*_act_, which could potentially span many orders of magnitude.

The probability of a pocket with a particular energy barrier being overcome at a particular time is given by 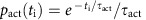. Once the energy barrier associated with a pocket has been overcome, let the volume recovered from that pocket vary with time according to 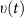
=*V*_0_Ф(*t*), which increases from zero to a maximum of, *V*_0_ over a time τ_h_.

We can write the total recovered volume at a given time in terms of an ensemble average over all the gas pockets of the probable amount of gas extracted from a given pocket at that time, given by equation (5).

Given a distribution of activation times, *p*_*τ*_, we can write equation (5) as an integral:


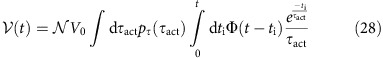


To explore the scaling of gas recovery with time according to equation (28), we assume an exponential distribution of energy barriers:


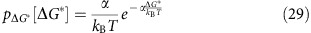


This simply represents one possible distribution, and the precise form is not critical to our analysis. The corresponding distribution of activation times is


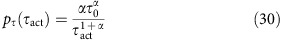


where we define 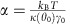; *θ*_0_ and *γ*_0_ fix the typical range spanned by these parameters over the reservoirs.

Taking this distribution of barriers and substituting into equation (28), we find the expression


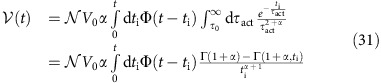


where Γ(*α*,*t*) is the incomplete gamma function. The solution to this expression is obtained for the two regimes *t*<*τ*_h_ and *t*>*τ*_h_ by using the Laplace transform for the convolution to get the asymptotics, with the results 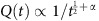 and 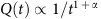, respectively, as discussed above.

### Data availability

The data that support the findings of this study are available from the corresponding author on request.

## Additional information

**How to cite this article:** Lee, T. *et al*. Activated desorption at heterogeneous interfaces and long-time kinetics of hydrocarbon recovery from nanoporous media. *Nat. Commun.* 7:11890 doi: 10.1038/ncomms11890 (2016).

## Supplementary Material

Supplementary Information Supplementary Figures 1-7, Supplementary Tables 1-4, Supplementary Note 1, Supplementary Discussion and Supplementary References.
