# Supplementary material for: Activated desorption at heterogeneous interfaces and long-time kinetics of hydrocarbon recovery from nanoporous media
Source: Nat Commun. 2016 Jun 21;7:11890. doi: 10.1038/ncomms11890 (PMC4919511; doi:10.1038/ncomms11890)
Supplement: Supplementary Information — Supplementary Figures 1-7, Supplementary Tables 1-4, Supplementary Note 1, Supplementary Discussion and Supplementary References. [file ncomms11890-s1.pdf]

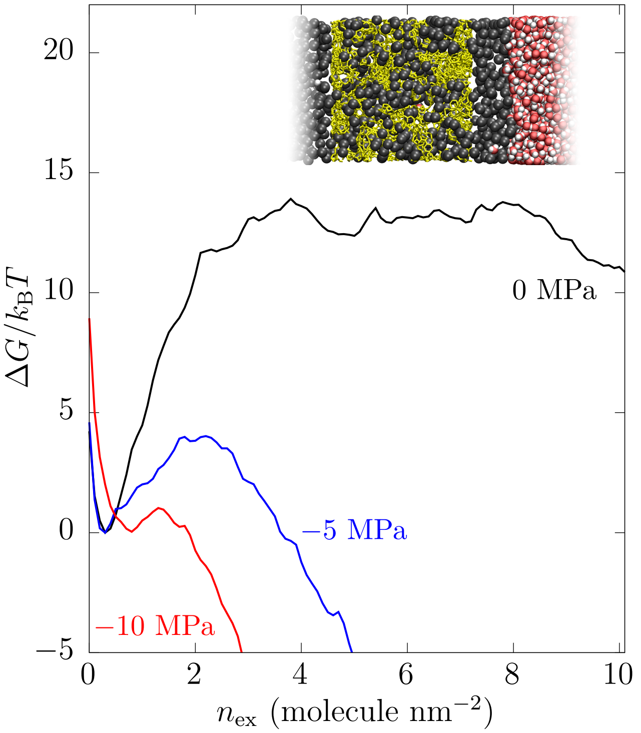

Supplementary Figure 1. **Activated recovery from a disordered nanoporous membrane.** Free energy $\Delta G/k_{\mathrm{B}}T$ measured using umbrella sampling as a function of extracted methane n_{ex} for the disordered porous carbon membrane pictured in the upper left inset. The free energy for $\Delta P = 0$ and two additional pressure differences are shown. The free energy results are qualitatively similar to those observed for the carbon nanotube membrane. This demonstrates the generality of the effect, which applies to non-ideal structures. The increase in free energy was smaller due to the greater porosity of the disordered membrane.

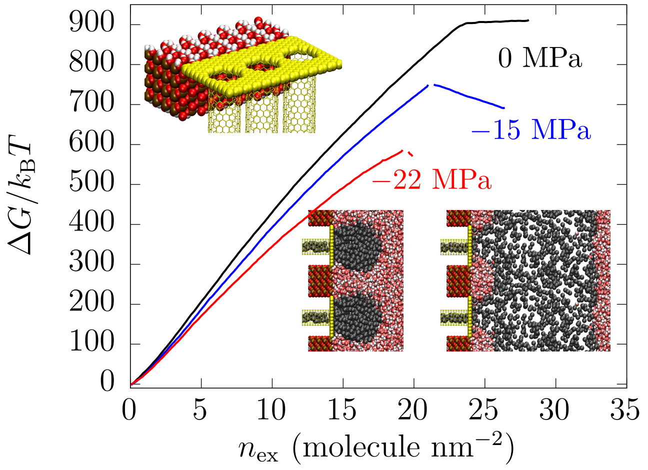

Supplementary Figure 2. **Activated recovery from a heterogeneous membrane.** Free energy $\Delta G/k_{\mathrm{B}}T$ measured using umbrella sampling as a function of extracted methane n_{ex} for a composite membrane consisting of a porous organic region and hydrophilic quartz region – pictured in the upper left inset. The free energy for $\Delta P = 0$ and two additional pressure differences are shown. The lower right insets show two periodic images of the system before and after the maximum free energy at $\Delta P = 20$ MPa. The free energy increased far more rapidly than for the pure organic membrane. Once the organic component is completely covered by methane, the methane phase extends out into the water phase, in the manner of a bubble on a hydrophilic surface, rather than covering the quartz. This behavior, shown in the lower-right inset, is an expected consequence of the strong adsorption of water at the quartz surface. At the point where the nucleus becomes large enough to stretch over the hydrophilic region and combine with its periodic image, as illustrated in the inset, the free energy plateaus when $\Delta P = 0$ and peaks when $\Delta P < 0$. Water drops remained adsorbed to the quartz even after this critical point.

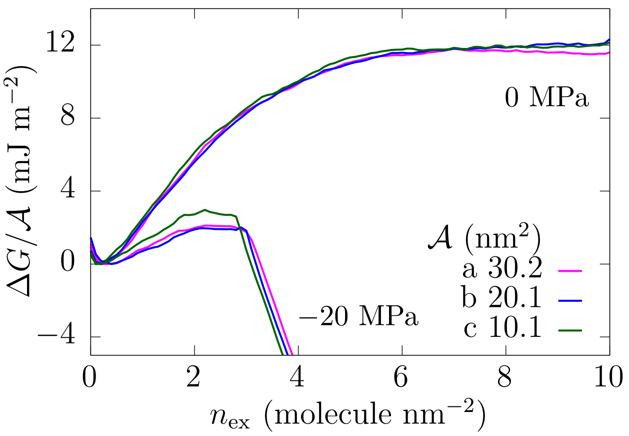

Supplementary Figure 3. **Free energy changes invariant with system size.** The free energy per unit area measured using umbrella sampling as a function of extracted methane in systems a, b, and c (Supplementary Table 3), which differ only in system size. Two pressure differences are shown, indicated in the figure. We see very close agreement between the different systems for all n_{ex} when $\Delta P = 0$, confirming the proportionality with the cross-sectional area $\mathcal{A}$ in equation 2 in the article. When $\Delta P < 0$ the agreement still holds approximately, with some small deviations. These deviations are likely to be a result of the constraint placed on the nucleus created by the periodic boundary conditions.

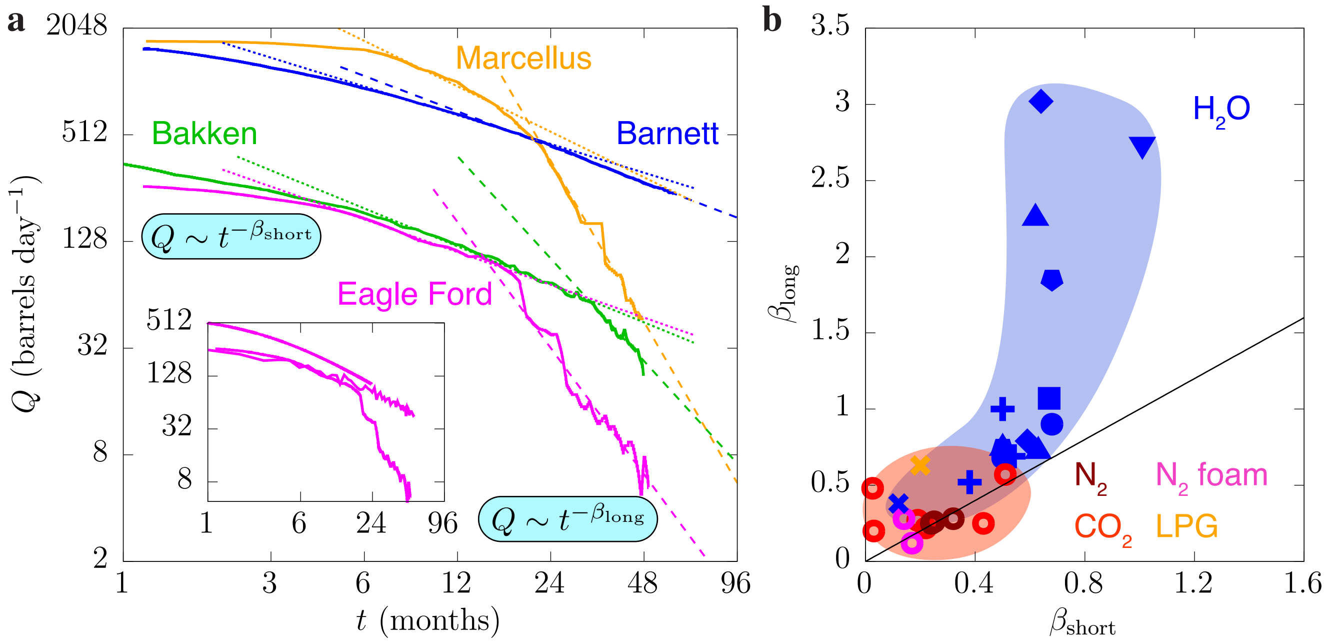

Supplementary Figure 4. **Production decline in unconventional reservoirs.** **a**, Typical examples of gas production over time for four wells in different gas-producing regions ("plays") in the United States[1]. Characteristic features include an initial transient period lasting several months; a second regime consistent with an algebraic decay $Q(t) \sim t^{-\beta_{\text{short}}}$ (dotted lines), and later regime with a larger decline exponent $Q(t) \sim t^{-\beta_{\text{long}}}$ (dashed lines). The inset shows gas production from several wells within the Eagle Ford region[1–3]. **b**, A comparison of the decline exponents at short and long times for various wells in different regions of the United States and Canada (marker shape) and with different fracking fluids (marker color). The solid line corresponds to $\beta_{\text{short}} = \beta_{\text{long}}$. Markers correspond to: squares, Fayatteville[4]; filled circles, Woodford[2, 4]; triangles, Eagle Ford[1–3]; inverted triangles, Haynesville[1]; diamonds, Marcellus[1, 3]; pentagons, Bakken[1, 2]; +, Barnett[1, 2, 4]; ×, New Brunswick[5]; open circles, Eastern Kentucky[6].

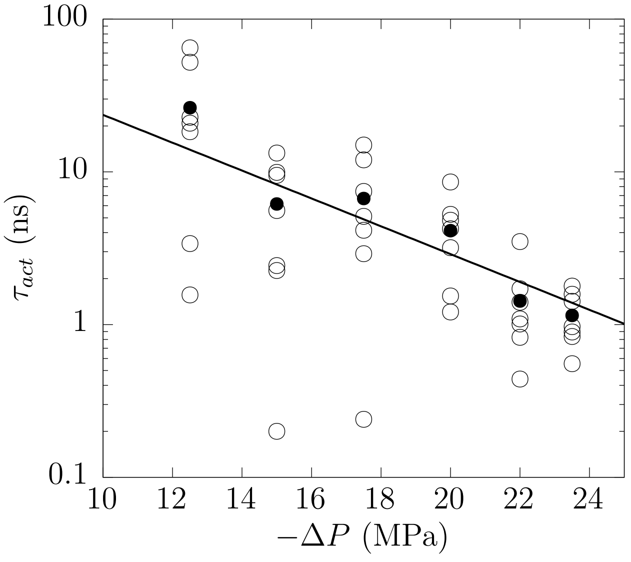

Supplementary Figure 5. **Activated extraction of methane from pores under applied pressure difference.** The average time $\tau_{\rm act}$ until an escape event (filled circles) as a function of the applied pressure difference ΔP, for a membrane with a pore radius $r = 0.59$ nm and pore spacing $D = 1.70$ nm, and lateral dimensions 1.704 nm $\times$ 2.951 nm. The open circles indicate the results for individual trials used for the averaging. The solid line is an exponential fit to the mean values. While there is considerable scatter in the individual trials at a given pressure – as expected for an activated process – the mean values are clearly consistent with an exponential trend. Despite such an expected and significant scattering, other functional forms would not allow fitting/rationalizing this data. More importantly, the activated nature of transport across hydrocarbon/water interfaces in shales is demonstrated in the article using a multiscale approach which culminates with rigorous account for variable and fast declines observed in shale gas productivity.

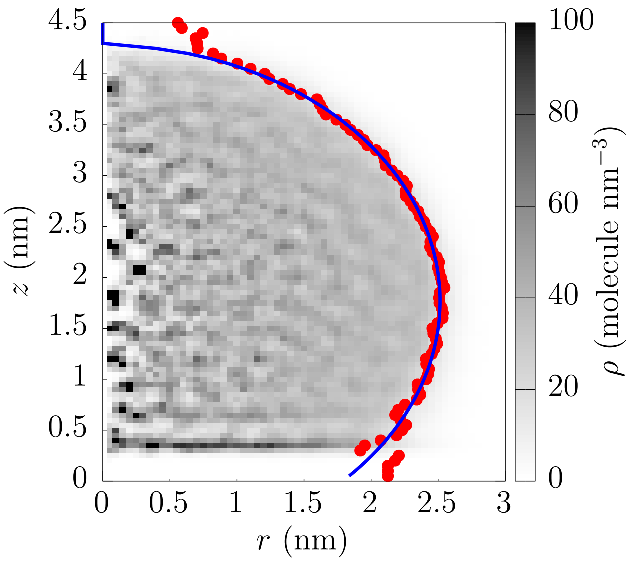

Supplementary Figure 6. **Contact angle of a water drop.** The density of a water drop on the model graphene surface as a function of the radial r and surface normal z coordinates. The red markers represent the position r_s of the liquid-vapour interface of the drop as determined by fitting the average density as a function of r within slabs 0.5 Å thick in the z direction. The fit is to a sigmoidal function $\rho(r) = \frac{\rho_L}{2} \tanh\left(2(r - r_s(z))/w\right)$ *via* r_s, the position of the interface, and ρ_L, with $w = 1$ Å. The blue line represents a circular fit to the interface as determined by the red markers. The fitted contact angle is 133°, confirming the hydrophobic nature of the membrane. While this is greater than the approximately 90° angle observed in experiments and in simulations using more detailed models[7], we aim only to model a simplified hydrophobic membrane, which this system provides.

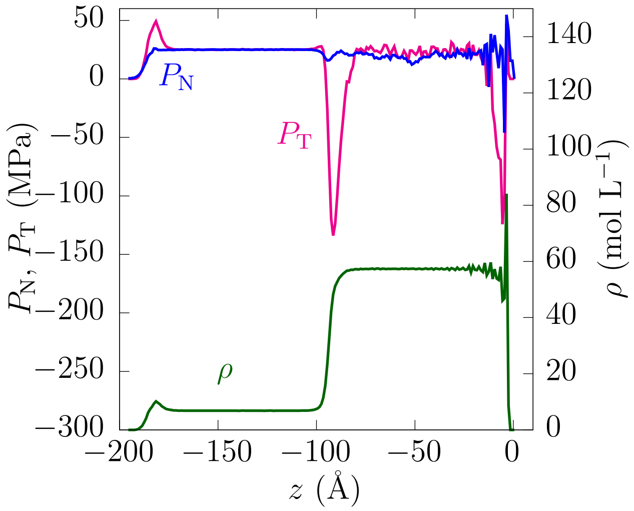

Supplementary Figure 7. **Surface tension from normal and tangential pressure profiles.** Density (ρ) and normal and tangential pressure (P_N, P_T) in the methane–water system used to measure the surface tension of the methane–water interface. These measurements indicate that the surface tensions of the interfaces are $\gamma_{MA} = -15.8$ mJ m^{-2}, $\gamma_{MW} = 82 \pm 4$ mJ m^{-2}, and $\gamma_{AW} = 116 \pm 4$ mJ m^{-2}. The corresponding spreading parameter is $\mathcal{S} = -18 \pm 8$ mJ m^{-2}. The good agreement with the result $\mathcal{S} = -16.55 \pm 0.41$ mJ m^{-2} as measured in the umbrella sampling simulations supports our assertion that the energy barrier is a result of a net increase in interfacial tension. The spreading parameter as measured by the pressure tensors should be slightly greater than in the umbrella sampling, as in the metastable state depicted in Fig. 3b(I), we expect a small amount of dissolved methane to lower the graphene–water surface tension. The negative value of the graphene–methane surface tension is a consequence of the fact that the atoms in the pistons are frozen in the x-y plane, and so do not contribute to the pressure tensor.

2. SUPPLEMENTARY TABLES

Supplementary Table 1. Fitted and predicted values of the effective contact angle for the results shown in Fig. 4 of the article.

ϕ type	θ_{eff} Fit	θ_{eff} Cassie-Baxter
0.35	25.0	26.0
0.58	19.7	20.9
0.75	14.3	16.1

Supplementary Table 2. Lennard-Jones parameters, charge, and mass. Lennard-Jones forces are truncated at a separation $r_{\mathrm{c}} = 13.5$ Å. There are two exceptions: for the interaction between piston particles and others, forces are truncated at the minimum in the Lennard-Jones potential, making interactions purely repulsive; and for the piston–methane interaction $\sigma_{ij} = 6.4$ Å to prevent gradual methane accumulation at the piston on the water phase during umbrella sampling.

type	ϵ (kJ/mol)	σ (Å)	charge (e)	mass (g/mol)
CH_4	1.23054	3.73	0	16
H (H_2O)	0	N/A	0.41	1
O (H_2O)	0.64978	3.166	−0.82	16
O (CO_2)	0.66937	3.033	−0.3256	16
C (CO_2)	0.23388	2.757	0.6512	12
C (membrane, piston)	0.23280	3.4	0	12

Supplementary Table 3. Geometry of the different membranes used in umbrella sampling simulations. r and D are the pore radius and pore separation respectively (relative to the carbon atoms centers), and N_x and N_y are the number of pores in each row of the triangular lattice and number of rows respectively.

system	r (nm)	D (nm)	N_x	N_y
a	0.587	1.704	3	4
b	0.587	1.704	2	4
c	0.587	1.704	2	2
d	0.587	2.130	2	2
e	0.587	2.556	2	2
f	0.587	2.982	2	2
g	0.705	2.982	2	2
h	0.822	2.982	2	2

Supplementary Table 4. Lennard-Jones parameters and charge for the atoms from which the quartz section of the composite membrane is composed. Mass is not tabulated, as it is irrelevant for frozen atoms.

type	ϵ (kJ/mol)	σ (Å)	charge (e)
Si	0.64852	3.7950	2
O	0.53346	3.1540	-1
H	0	N/A	0.5

3. SUPPLEMENTARY NOTES

Supplementary Note 1. We employed the following minimization algorithm within the *Surface Evolver* program to find the minimum-energy droplet shape on an idealized nanoporous interface. At the end of this process, no combination of minimization steps results in any significant decrease in the surface energy of the system.

```
repeat
    Run linear gradient decent
until Scale factor equals zero
repeat
    Run Hessian seek decent
until Scale factor is less than 1 × 10^-13
for 3 iterations do
    Refine facets
    repeat
        Freeze the contact line
        repeat
            Run Hessian seek minimization
        until Scale factor equals zero
        Run 20 iterations of linear gradient decent
        Unfreeze the contact line
        repeat
            Run 5 iterations of linear gradient decent
            repeat
                Run Hessian seek minimization
            until Scale factor is less than 1 × 10^-13
            repeat
                Run Hessian seek minimization with a maximum scale factor 2 (default 1)
            until Scale factor is less than 1 × 10^-13
            Run 5 iterations of linear gradient decent
        until Scale factor equals zero
        Freeze the contact line
```

repeat

Run Hessian seek minimization

until Scale factor equals zero

Unfreeze the contact line

Run 1 interaction of linear gradient decent

until Scale factor equals zero

end for

End

4. SUPPLEMENTARY DISCUSSION

We have gathered data from publicly available literature on gas production over time for some typical examples of unconventional wells from different shale plays [1]. Results are shown in Supplementary Figure 4a. The very limited number of studies accessible to the scientific community concern some vertical wells in addition to the more common horizontal wells and include both shale oil and shale gas wells. However, despite such data scarcity and variety, a general kinetic behavior of shale gas extraction can be established from Supplementary Figure 4. After an initial transient period during the first few months of production, wells tend to settle into a first algebraic decay $Q(t) \sim t^{-\beta_{\text{short}}}$, indicated by the dotted lines in Supplementary Figure 4a, with β_{short} typically less than unity and sometimes but not generally close to 1/2. Such a rather slow algebraic decay can be rationalized on the basis of Darcy-like framework, as *e.g.* reported by Patzek *et al.* [8] and Monteiro *et al.* [9]. This leads to a $Q(t) \sim t^{-1/2}$ decay rate, as expected from boundary limited flow. Such models predict an exponential decay at long time, associated with full depletion of the gas region. However, as shown in Supplementary Figure 4a, data suggest that for long times, production enters a second regime, rather consistent with an algebraic decay $Q(t) \sim t^{-\beta_{\text{long}}}$. This is shown in Supplementary Figure 4a as dashed lines, in most cases with a far more rapid decline $\beta_{\text{short}} < \beta_{\text{long}}$. While the short and long time range productions have been assigned respectively to Darcy-limited and transport-limited regimes [8, 10], the physical origin and effects of well specificities (including their interfacial physical chemistry) for such a transition to a more rapid rate with algebraic decline remain unclear. For instance, large variations in behavior are observed even within a given geographic region, such as the Eagle

Ford basin [1–3] shown in the inset to Supplementary Figure 4a. Furthermore, some wells such as those from the Barnett region, known for their excellent productivity and relatively long history, are somewhat atypical in that the gas production generally declines relatively slowly compared to other regions with a weakly pronounced difference between the short and long time ranges and a decay exponent close to that expected for a boundary-limited flow ($\beta = 1/2$).

In order to highlight this two-regime scenario, we gather in Supplementary Figure 4b the measured β exponents at early and late times as a parametric plot, for a number of decline curves reported in the literature [1–6]. For hydrofracked wells (blue markers) for which a water-sand mixture is used as the pressure transmitting fluid, the β exponent is always larger at longer times with an overall tendency for larger β_{short} to correlate with larger β_{long}. Although most unconventional wells are stimulated using a water-based fracking fluid, waterless or low-water fluids have also been used in some cases including liquid petroleum gas (LPG) [5], supercritical CO_2, nitrogen gas, and nitrogen-water foams [6]. Our survey of the literature suggests that wells fracked using such fluids tend to have smaller decline exponents, occupying the red-shaded region of Supplementary Figure 4b. Non-water fluids have some disadvantages, most importantly an inferior ability to carry proppant particles to prevent fracture closure after stimulation, leading to lower total recovery.

5. SUPPLEMENTARY REFERENCES

---

[1] Hughes, J. D. Drill, baby, drill: Can unconventional fuels usher in a new era of energy abundance? Post Carbon Institute (2013).

[2] Dutta, R., Meyet, M., Burns, C. & Van Cauter, F. Comparison of empirical and analytical methods for production forecasting in unconventional reservoirs: Lessons from North America. *SPE/EAGE European Unconventional Resources Conference and Exhibition* 167734 (2014).

[3] Carrizo Oil & Gas, Inc. Wells fargo. In *5th Annual Exploration and Production 1X1 Forum* (Carrizo-2014).

[4] Baihly, J. D., Altman, R. M., Malpani, R. & Luo, F. Shale gas production decline trend

comparison over time and basins. *SPE Annual Technical Conference and Exhibition* 135555 (2010).

[5] Leblanc, D. P., Martel, T., Graves, D. G., Tudor, E. & Lestz, R. Application of propane (LPG) based hydraulic fracturing in the McCully gas field, New Brunswick, Canada. *SPE North American Unconventional Gas Conference and Exhibition* 144093 (2011).

[6] Mazza, R. Liquid-free CO_2/sand stimulations: An overlooked technology – production update. *SPE Eastern Regional Meeting* 72383 (2001).

[7] Taherian, F., Marcon, V., van der Vegt, N. F. A. & Leroy, F. What is the contact angle of water on graphene? *Langmuir* **29**, 1457–1465 (2013).

[8] Patzek, T. W., Male, F. & Marder, M. Gas production in the Barnett shale obeys a simple scaling theory. *Proc. Natl. Acad. Sci. U.S.A.* **110**, 19731–19736 (2013).

[9] Monteiro, P. J. M., Rycroft, C. H. & Barenblatt, G. I. A mathematical model of fluid and gas flow in nanoporous media. *Proc. Natl. Acad. Sci. U.S.A.* **109**, 20309–20313 (2012).

[10] Silin, D. & Kneafsey, T. J. Shale gas: Nanometer-scale observations and well modelling. *J. Can. Petrol. Technol.* **51**, 464475 (2012).
